# The peripheral and decidual immune cell profiles in women with recurrent pregnancy loss

**DOI:** 10.3389/fimmu.2022.994240

**Published:** 2022-09-13

**Authors:** Dengke Qin, Huihui Xu, Zechuan Chen, Xujing Deng, Shan Jiang, Xiaoming Zhang, Shihua Bao

**Affiliations:** ^1^ Department of Reproductive Immunology, Shanghai First Maternity and Infant Hospital, School of Medicine, Tongji University, Shanghai, China; ^2^ Shanghai Key Laboratory of Maternal Fetal Medicine, Shanghai Institute of Maternal-Fetal Medicine and Gynecologic Oncology, Shanghai First Maternity and Infant Hospital, School of Medicine, Tongji University, Shanghai, China; ^3^ The Center for Microbes, Development and Health, Key Laboratory of Molecular Virology & Immunology, Institut Pasteur of Shanghai, Chinese Academy of Sciences/University of Chinese Academy of Sciences, Shanghai, China

**Keywords:** recurrent pregnancy loss, peripheral blood, decidual tissue, flow cytometry, immune profile

## Abstract

Recurrent pregnancy loss (RPL) affects 1-2% of couples of reproductive age. Immunological analysis of the immune status in RPL patients might contribute to the diagnosis and treatment of RPL. However, the exact immune cell composition in RPL patients is still unclear. Here, we used flow cytometry to investigate the immune cell profiles of peripheral blood and decidual tissue of women who experienced RPL. We divided peripheral immune cells into 14 major subgroups, and the percentages of T, natural killer T (NKT)-like and B cells in peripheral blood were increased in RPL patients. The decidual immune cells were classified into 14 major subpopulations and the percentages of decidual T, NKT-like cells and CD11c^hi^ Mφ were increased, while those of CD56^hi^ decidual NK cells and CD11c^lo^ Mφ were decreased in RPL patients. The spearmen correlation analysis showed that the proportion of peripheral and decidual immune cells did not show significant correlations with occurrences of previous miscarriages. By using flow cytometry, we depicted the global peripheral and decidual immune landscape in RPL patients. The abnormalities of peripheral and decidual immune cells may be involved in RPL, but the correlations with the number of previous miscarriages need further verification.

## Introduction

Recurrent pregnancy loss (RPL) is usually defined as the consecutive loss of two or more pregnancies before 24 weeks gestation, which affects about 1 to 2% of couples all over the world (1). The etiology of RPL is extremely varied which includes genetic abnormalities, uterine anomalies, endocrine dysfunction, parental balanced chromosomal translocation and infections (2). However, more than 50% of RPL patients can not be found obvious causes even after a comprehensive investigation, which is often called unexplained RPL (uRPL) (3). It has been proposed that most of the uRPL cases could attribute to immune system dysregulation, which is usually referred to as immune-related RPL (4).

During pregnancy, the fetus is a semi-allogenic to the maternal host, the maternal-fetal immune tolerance plays a key role in establishing a successful pregnancy (5). Maintaining new homeostasis of the maternal immune system is essential for ensuring maternal tolerance (6). Given the key function of immune cells in pregnancy, studies on immune-related causes of RPL have attracted great attention. For example, some researchers focus on the disorders of peripheral blood immune cells in RPL, given the difficulties of the invasive uterine specimen. The unbalance of Th1/Th2/Th17 and Treg cell paradigm could contribute to pregnancy complications like preeclampsia (PE) and RPL (7). Some studies have shown that the elevated levels and activation of CD3^+^CD56^+^ NKT-like cells in peripheral blood could lead to poor pregnancy outcomes by activating leukocyte subsets and inducing trophoblast cell death (8, 9). Peripheral NK (pNK) cells were phenotypically and functionally different from the decidual NK cells (10). The higher pNK cell activity might be a biomarker for predicting subsequent miscarriage, but the accuracy still needs further verification (11). In addition, the higher ratio of peripheral polymorphonuclear leukocytes (PMN) and monocytes indicated a possible association with inflammation state during pregnancy (12).

Decidual immune cells are composed of about 30-40% decidual cells during early pregnancy, the abundance and subtype of decidual immune cells have been confirmed associated with pregnancy complications (13, 14). The aberrations in the number or differentiation of decidual T and NK cells may induce serious dysfunction of the reproductive system, including RPL (15, 16). The inappropriately differentiated macrophages have been shown to leading reproductive complications including RPL, preeclampsia (PE), and intrauterine growth restriction (IUGR) (5, 17). Decidual PMNs and DCs may also have been implicated in both the initiation and later pathogenesis of RPL (18–20).

Though a few studies used single-cell RNA sequence and immunohistochemistry (IHC) analysis to explore the immune cell features in RPL patients, they were not robust quantitative methods for cell populations (14, 16). The global peripheral and decidual immune landscape in RPL patients is still unclear. Therefore, it is an urgent need to develop a reliable method to characterize the peripheral blood and decidual immune cell populations in RPL patients to predict and evaluate the disease progression. Herein, we used eighteen-color flow cytometry to establish different immune cell compositions in peripheral blood and decidual tissues from RPL patients. To make the experiments readily accessible to other researchers, we offered the detailed established antibody panels and the gating strategies to identify and quantify the various immune cells. Our research may contribute to a better understanding of the immune cell profiles in RPL, as well as identifying potential pathomechanisms and therapeutic targets for future explorations.

## Material and methods

### Human samples

Blood and decidual tissues were obtained in the first trimester from HDs in normal pregnancy (n = 23) for selective termination or RPL patients (n = 21) who met the diagnostic criteria of RPL were included (21). All enrolled samples received administration of 400µg misoprostol vaginally two hours before abortion surgery. Normal peripheral blood and decidual specimens with no previous abortions were collected from elective terminations of normal pregnancies. Patients with two or more consecutive previous spontaneous unexplained miscarriages, using Chromosomal Microarray Analysis to detect fetal chromosomal abnormalities. The normal karyotype of parents and abortus were enrolled in the RPL cohort. Patients with implantation failure due to anatomic, hormonal, infectious, autoimmune, or thrombosis-based causes were excluded from the study group. No participants had a history of smoking or alcohol use. The detailed clinical characteristics of the enrolled donors were summarized in **Table 1**. All samples in this study have signed written informed consent. This study was approved by the Research Ethics Committee in the Hospital of Shanghai First Maternity and Infant Hospital (Shanghai, China).

**Table 1:** Clinical characteristics of HDs and RPL patients.

Variables	HD (n=23)	RPL (n=21)	P value
Ave age (year)	30.39±1.64	31.19±3.72	0.355
Ave No. of gravidities	1.65±0.83	3.67±1.93	<0.001
Ave No. of previous miscarriage	0	3.48±1.63	<0.001
Ave gestation (week)	6.74±0.45	6.81±0.40	0.588
Ave BMI (week)	21.03±1.34	22.67±4.14	0.077

Ave, Average; BMI, Body Mass Index.

### Peripheral blood collection and decidual cells isolation

Fasting peripheral blood samples were collected before the surgery. 1.5ml of whole blood was extracted for each flow cytometry staining panel.

Decidual tissues were transferred to the laboratory on ice in a DMEM medium with 10% fetal bovine serum (Gibco). Decidual tissues were washed extensively with 1x cold PBS (Gibco) to remove the blood as much as possible. To generate single-cell suspensions, the tissues were mechanically dissociated using scissors and digested for 45 min each at 37°C with a medium containing 1 mg/mL collagenase IV (Gibcol, 17104019). The digested tissues were filtered through a 40-μm cell-strainer nylon mesh (BD) and were collected by centrifuging at 700 × g for 10 min. After removing the supernatant, the cell pellet was washed twice with Flow Cytometry Staining Buffer (FACS buffer). FACS buffer was composed of PBS containing 1% FBS, 0.5% EDTA, and 0.05% gentamycin.

### Flow Cytometry

A list of 17 monoclonal antibodies including their label, clone, source, dilution and staining information was provided in **Supplementary Table S3**. All data were carried out using BD FACS Fortessa. The normalized FCS files were analyzed by the FlowJo software (BD) and data was presented as a percentage of CD45^+^cells.

### Statistical analysis

Statistical calculations were performed with the R, SPSS (v22, IBM, Armonk, NY), and Prism 6.0 (SanDiego, CA) software. Comparisons were performed using the χ2 test, paired t-test, or two-sided Wilcoxon Rank Sum test as needed. All tests were 2-sided, and significance levels were set to p<0.05 (*), p<0.01 (**), p<0.001 (***), p<0.001 (****) and ns means no significance. Correlations between two variables were determined by using Spearman’s rank coefficient.

## Results

### Flow cytometric analysis of peripheral blood immune cells

**Figure 1:**
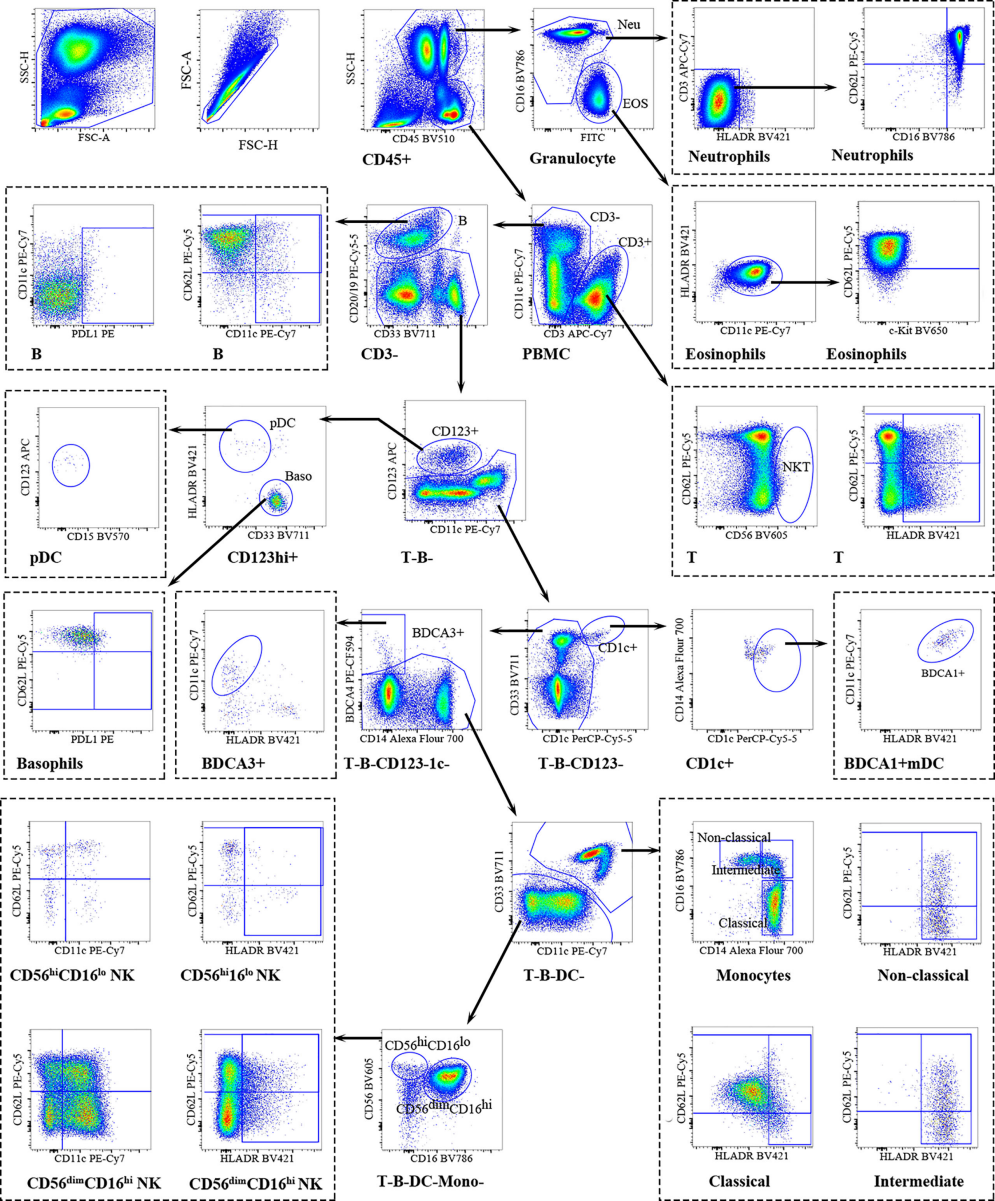
Overview of peripheral immune cells gating strategy. Representative polychromatic dot plots demonstrating the gating strategy used to identify peripheral immune cell content in RPL patients and HDs. All leukocytes were selected using CD45- positivity and then divided into separate cell subtypes as shown.

Using a detailed flow cytometry panel, we comprehensively assessed the immune cell composition in peripheral blood from RPL patients and HDs (**Figure 1**). CD45^+^ cells were further differentiated into neutrophilic and eosinophilic granulocytes (SSC^hi^) and PBMC (SSC^lo^). Peripheral blood neutrophils were homogeneous and express FcγRIII (CD16) and L-selectin (CD62L) within a narrow range, typically characterized as CD16^bright^CD62L^bright^ (mature), CD16^dim^CD62L^bright^ (immature), CD16^dim^CD62L^dim^ (apoptotic) neutrophils (22). L-selectin(CD62L) can account for impaired rolling and migration ability of leukocytes (23). The expression of HLA-DR was defined as an activation marker (24). Using CD62L and HLA-DR to characterize CD3^+^T cells into CD62L^+^ and HLA-DR^+^ CD3^+^T cells. CD56 was used for the identification of NKT -like cells, defined as CD56^+^CD3^+^T cells, which might serve as a bridge between innate and adaptive immunity. CD19 and CD20 were chosen for the identification of B cells. Plasmacytoid dendritic cells (pDC, HLA-DR^+^CD123^+^) and basophils (HLA-DR^-^CD123^+^) were selected from CD3^-^CD20/19^-^ cells. CD1c^+^ conventional DC2 (BDCA1^+^-DCs) and BDCA3/CD141^+^ conventional DC1 (BDCA3^+^-DCs) were gated the from the remaining cells (T, B, pDC, basophils). Monocytes were identified based on the expression of CD14 and were subsequently divided into three subsets; classical (CD14^++^CD16^−^), intermediate (CD14^++^CD16^+^), and nonclassical monocytes (CD14^+^CD16^++^). pNK cells were gated into the early pNK (CD56^hi^CD16^lo^) and mature pNK (CD56^dim^CD16^hi^) subsets. The activation status of pNK cells was monitored by regulating the expression of HLA-DR. CD62L and CD11c respectively represented the adhesion ability and effector function capacity and they were used to divide pNK cells into subgroups (25, 26).

### Global changes in peripheral blood immune cell profiles

**Figure 2:**
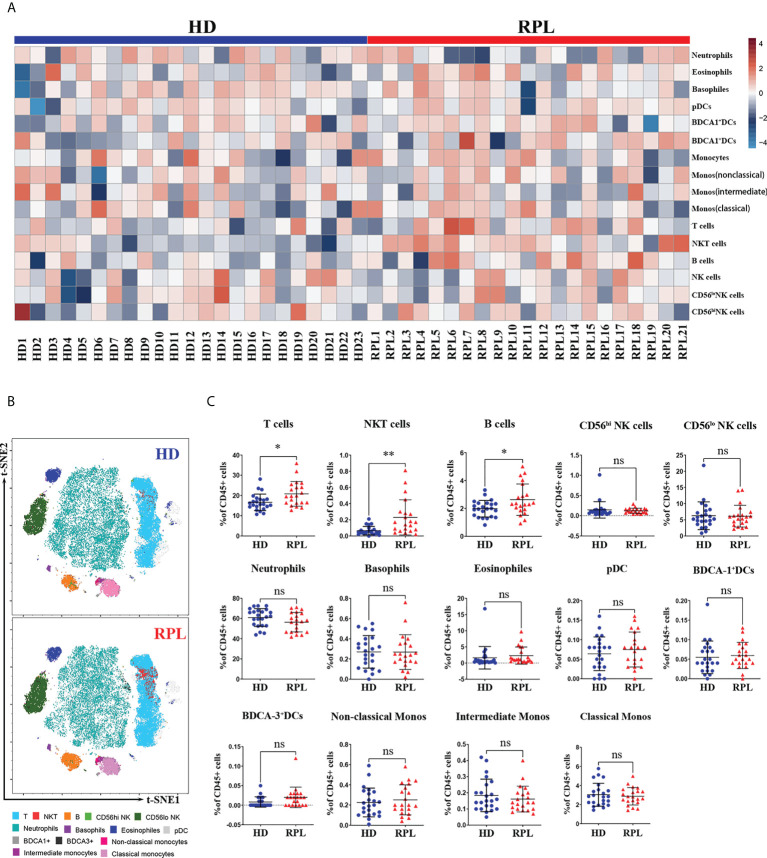
Global changes in peripheral immune cell profiles. **(A)** Heatmap representation presenting the patient-to-patient variability of the log odds ratio derived from the percentage of CD45^+^ cells. **(B)** Representative tSNE plots of clustered CD45^+^ peripheral immune cells were shown changes in RPL patients and HDs. **(C)** Regulated peripheral immune cell populations differentially abundant between RPL patients and HDs identified by flow cytometry. p<0.05 (*), p<0.01 (**) and ns, no significance.

We sought to determine whether RPL patients and HDs peripheral blood show distinct immune cell signatures. In HDs, 66.54% of all CD45^+^cells were from the myeloid lineage, predominantly neutrophils (60.92%), monocytes (3.65%) and eosinophils (1.58%); and 27.29% of the lymphoid lineage, primarily T-cells (16.83%), pNK cells (8.50%) and B cells (1.97%). Minor populations included dendritic cells (DCs) and basophils. In RPL patients, the distribution of peripheral blood immune cells was different. The proportion of myeloid cells was reduced to 62.58%, mainly neutrophils (56.13%), monocytes (3.63%) and eosinophils (2.40%). The lymphoid-derived cells were enriched to 31.71%, principally T-cells (20.78%), NK cells (8.26%) and B cells (2.68%) (**Table S1**). Using heatmap representations we examined how the distribution of the percentage of CD45^+^ cells of each cell type varied in every patient. With each group, relatively homogeneous patterns were observed. The differences between RPL patients and HDs were well-defined as highlighted by the enrichment of T, NKT-like and B-cells (**Figure 2A**).

To gain a broad overview of the peripheral blood immune cell populations, we performed t-SNE analysis on CD45^+^ cells from RPL patients and HDs (**Figure 2B**). Compared to HDs, RPL patients had an increased fraction of CD3^+^T cells and NKT-like cells (**Figure 2C**). Furthermore, we observed a significant increase in B cells and CD62L^+^B cells (**Supplementary Figure 1A**). Unlike other studies, the percentage of CD56^hi^ and CD56^lo^ pNK cells and subsets were not significantly different between RPL patients and HDs (**Figure 2C**). Meanwhile, there were no differences in the proportions of neutrophils, basophils, eosinophils, DCs and monocytes (**Figure 2C**).

### Flow cytometric analysis of decidual immune cells

**Figure 3:**
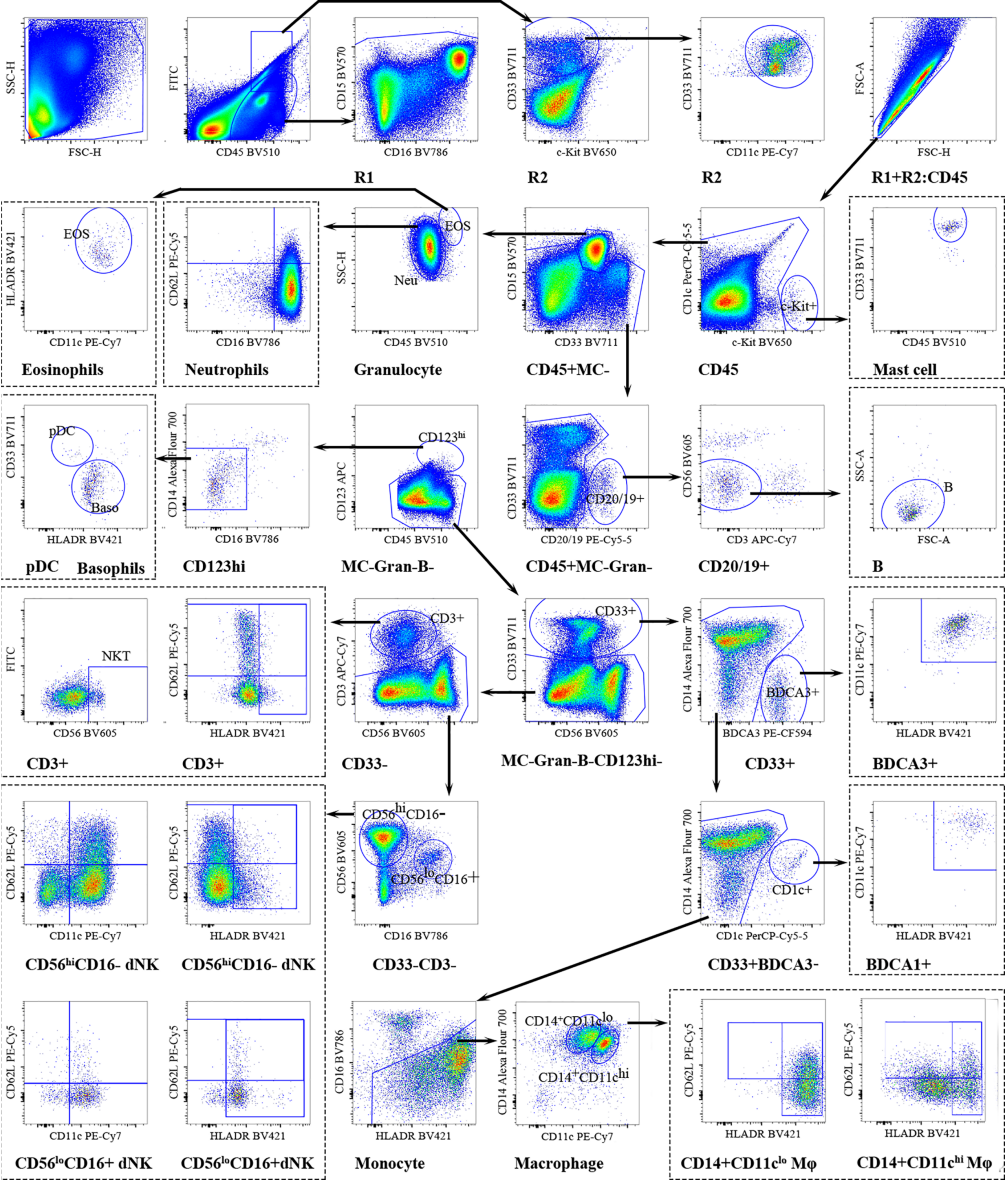
Overview of decidual immune cells gating strategy. Representative polychromatic dot plots demonstrating the gating strategy used to identify decidual immune cell content in RPL patients and HDs. All leukocytes were selected using CD45-positivity and then divided into separate cell subpopulations as shown.

The number of decidual CD45^+^ cells was calculated as lymphoid cells (R1) + myeloid cells (R2) (**Figure 3**). Mast cells (MCs) were identified by the expression of c-Kit. CD15 was chosen for the identification of neutrophils and eosinophils. Same as peripheral blood neutrophils, decidual neutrophils also characterized as CD16^bright^CD62L^bright^ (mature), CD16^dim^CD62L^bright^ (immature), CD16^dim^CD62L^dim^ (apoptotic) by CD16 and CD62L. CD19/20 were used to identify B cells, CD56 and CD3 were exclusively on NK- and T cells. To classify the basophils and pDCs, CD123 and HLA-DR were used as previously described. Basophils were identified by HLA-DR^-^CD123^+^; pDCs were identified as HLA-DR^+^CD123^+^cells. Conventional DCs were further subset into BDCA3^+^-DCs and BDCA1^+^-DCs based on BDCA3/CD141 and CD1c expression. Decidual macrophages were identified by CD14 and HLA-DR. Using CD11c and CD14, decidual macrophages could be further subsetted into CD11c^hi^ and CD11c^lo^ Mφ. CD62L and HLA-DR were used to evaluate migration and activation ability. On decidual CD3^+^T cells, CD56 was further used for the identification of NKT-like cells. Decidual NK (dNK) cells have been typically defined as CD56^hi^ CD16^-^ cells, distinct from peripheral blood NK cells (pbNK) and other tissue-resident NK cells (trNK) (27). The activation status of dNK cells was monitored by regulating the expression of HLA-DR. CD62L and CD11c were used to evaluate the adhesion ability and effector function capacity of dNK cells.

### Global changes in decidual immune cell profiles

**Figure 4:**
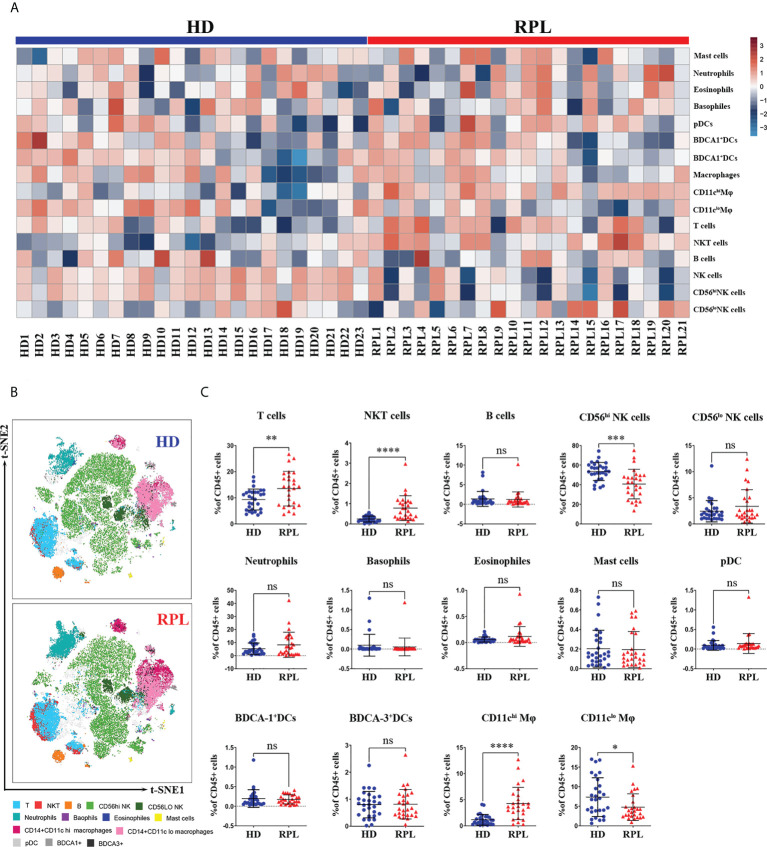
Global changes in decidual immune cell profiles. **(A)** Heatmap representation presenting the patient-to-patient variability of the log odds ratio derived from the proportion of CD45^+^ cells. **(B)** Representative tSNE plots of clustered CD45^+^ decidual immune cells were shown changes in RPL patients and HDs. **(C)** Regulated decidual immune cell populations differentially abundant between RPL patients and HDs identified by flow cytometry. p<0.05 (*), p<0.01 (**), p<0.001 (***), p<0.001 (****) and ns, no significance.

The decidual immune cell compositions in RPL patients and HDs were also varied. In HDs, 16.75% of all CD45^+^cells were from the myeloid lineage, predominantly macrophages (10.32%) and neutrophils (5.12%); and 66.30% of the lymphoid lineage, primarily dNK cells (55.36%) and T cells (9.34%). In RPL patients, the predominance of myeloid cells was increased to 19.25%, mainly macrophages (10.08%) and neutrophils (7.86%). The percentage of lymphoid-derived cells was reduced to 58.54%, principally dNK cells (43.20%) and T cells (13.97%). Minor populations included dendritic cells (DCs), basophils, eosinophils and B cells (**Table S2**). The expression level of different decidual immune cells of CD45^+^ cells in every sample was shown by the heatmap (**Figure 4A**).

The decidual immune cell populations were then mapped on t-SNE composite plots which showed a great overview of cellular changes in RPL patients and HDs (**Figure 4B**). The altered decidual lymphoid immune cell state in RPL patients was demonstrated by an increased number of T and NKT-like cells (**Figure 4C**). In addition, the number of CD56^hi^ dNK cells and HLA-DR^+^CD56^hi^ dNK cells was decreased in RPL patients. The myeloid-derived CD11c^hi^ Mφ, including both HLA-DR^+^ and CD62L^+^ subpopulations, were also significantly increased in RPL patients (**Figure 4C**; **Supplementary Figure 2A**). In HDs, the number of CD11c^lo^ Mφ was decreased, including both HLA-DR^+^ and CD62L^+^ subgroups (**Figure 4C**; **Supplementary Figure 2A**). Although statistical significance was not reached, a trend of increased neutrophils and eosinophils was observed in RPL patients (**Figure 4C**). No differences were observed in the proportion of the B cells, basophils, eosinophils and DCs (**Figure 4C**).

### The interrelationship of immune cells and correlation with clinical characteristics in RPL patients

**Figure 5:**
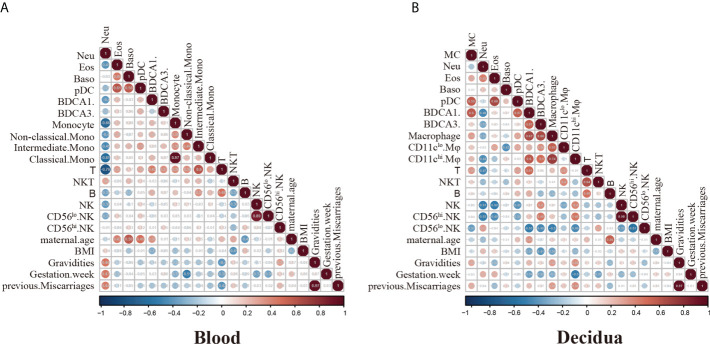
Spearman correlation analysis of immune cell frequencies and RPL patients’ clinical features. **(A)** Heatmap visualization shows spearmen correlation coefficient for clinical features including maternal age, BMI, gravidities, gestation week, BMI, previous miscarriages and peripheral immune cell frequencies. Key indicates r value scale for positive (red) and negative (blue) correlations. **(B)** Heatmap visualization shows spearmen correlation coefficient for clinical features including maternal age, BMI, gravidities, gestation week, BMI, previous miscarriages and decidual immune cell frequencies. Key indicates r value scale for positive (red) and negative (blue) correlations.

To better reveal the interrelationship of immune cells and correlation with clinical characteristics, we performed a spearman’s rank correlation coefficient test on the RPL patients’ cohort (n=21). The heatmap in **Figure 5A** showed all correlations between clinical data (maternal age, BMI, gravidities, gestation week, BMI, previous miscarriages) and peripheral immune cell percentage of the total CD45^+^cell compartment. Our data showed that neutrophils inversely correlated with the percentages of monocytes and T cells. Meanwhile, basophils and eosinophils positively correlated with pDCs. Although T, NKT-like and B cells increased in the peripheral blood of RPL patients, the statistics showed that there is no correlation with the number of previous miscarriages (**Figure 5A**). Notably, the peripheral neutrophils showed a positive correlation with the number of abortions, although this did not reach statistical significance (**Figure 5A**).

Concerning decidual immune cells, decidual T cells exhibited a positive correlation with NKT-like cells. Decidual pDCs showed a positive correlation with MCs, eosinophils and BDCA1^+^DCs. The expression of decidual macrophages was positively correlated with the BDCA1^+^DCs and BDCA3^+^DCs (**Figure 5B**). Though the number of decidual T, NKT-like and CD56^hi^ dNK cells has changed in RPL patients, the correlations with the numbers of miscarriages were not significant (**Figure 5B**). Our analysis showed the proportion of CD11c^hi^ Mφ has a positive correlation with miscarriage numbers, although the data did not exert statistically significant changes (**Figure 5B**).

## Discussion

Here, we utilized multi-color flow cytometry to create a systemic picture of the peripheral and decidual immune cell populations in RPL patients and HDs. Using this approach together with the concomitant bioinformatical analysis we have evaluated the presence and abundance of different peripheral and decidual immune cells. Compared to single-cell sequencing articles, flow cytometry could analyze and quantify multiple independent immune cell populations by using a large number of differentiation markers, including the multidimensional description of subpopulations and their activation or migration states. In our study, we have 1) identified and quantified a detailed picture of the peripheral and decidual immune phenotype in RPL patients and HDs; 2) evaluated cell populations that might contribute to the immune disorders in RPL patients; 3) assessed the interrelationships between immune cell populations and clinical characteristic in RPL patients.

Compared to HDs, the distribution and abundance of peripheral blood immune cell populations in RPL patients have changed. Similar to other studies, our data also showed that the number of T cells was increased in RPL patients, suggesting that the T cell abnormalities may be involved in RPL pathogenesis (28). The peripheral NKT-like cells could modulate the immune response of innate and adaptive immune cells by secreting inflammatory cytokines (29), we observed a strong increase in the number of NKT-like cells in RPL patients, implying that higher level of NKT-like cells might disturb the balance of the immune system. B cells were reported to play an important role in maintaining a successful pregnancy by regulating the secretion of autobodies and cytokines (30). Our results showed that the abundance of B and CD62L^+^ B cells was significantly higher in RPL patients, indicating that the alteration of immune effectors may be associated with RPL. CD11c^+^ B cells have been suggested to play a key play in various autoimmune diseases like graves’ disease, systemic lupus erythematosus (SLE) and Sjogren’s Syndrome (31). Programmed death-ligand 1 (PD-L1) is a critical molecule in immune suppression and the lower level of PD-L1^+^ B cells may also lead to poor pregnancy outcomes like RPL (32). However, there were no differences in the expression of CD11c^+^ and PD-L1^+^ B cells in RPL patients and HDs in this study. Consistent with the previous study pNK cells might not be an independent risk factor for subsequent miscarriages (33), we also demonstrated no significant differences between pNK cells and their subtypes in the present study. Though it was reported that the number of peripheral PMN has changed in RPL patients (34), our results showed that the number of granulocytes including neutrophils, basophils, eosinophils and their subpopulations was no different. Several studies revealed that DCs and monocytes might play a role in defending against pathogens and preventing the immune rejection of the fetus (35). Nonetheless, the percentages of three types DCs (pDCs, BDCA1^+^DCs and BDCA3^+^DCs) and monocytes (classical, intermediate, nonclassical) in RPL patients and HDs in our study did not show differences.

Moving from the global peripheral immune cell picture to decidual tissue, we also found the number and classification of decidual immune cells have changed in RPL patients. It has been reported that decidual T and NKT-like cells could accumulate in an antigen-nonspecific fashion at sites of inflammation including chronic deciduitis, chronic chorioamnionitis and chronic villitis/villitis of unknown etiology (VUE) (36). Similar to other studies, the number of decidual T and NKT-like cells in our study was also significantly increased in RPL patients (37). The aggregation of T and NKT-like cells in both peripheral blood and decidual tissues suggested that the RPL patients may be under a chronic inflammation status which leads to failed pregnancies. The subpopulations of T cells including HLA-DR^+^ and CD62L^+^ T cells did not increase in RPL patients, which suggests that the peripheral and decidual T cells of RPL patients may be in a low activation state (38). Unlike peripheral blood B cells, the number of decidual B cells was small and did not increase in RPL patients. Due to the high prevalence in the decidual uterine lymphocyte population, dNK cells have been classically associated with reproductive success by controlling trophoblast invasion and remodeling spiral arteries (39, 40). A recent systematic review has shown that there was no significant difference between dNK subtypes although subgroup analysis revealed notably higher total CD56^+^ uterine NK cells in RPL patients compared with HDs using endometrial specimens from the mid-luteal phase only. However, this observation was not successfully replicated by testing decidual tissue in first-trimester pregnancy (41). These results were in contrast to our study where we observed a decreased abundance of CD56^hi^ dNK cells in RPL patients, including HLA-DR^+^ CD56^hi^ and CD11c^-^CD62L^-^CD56^hi^ dNK cells. The cytotoxic CD56^lo^ dNK cells were increased in RPL patients, though the difference did not show statistical significance. We suggested that our findings were reflective of the increased accuracy of multicolor flow cytometry over IHC in quantitative studies; many of the 33 studies included in the meta-analysis of RPL patients were IHC studies only. In the present study, two distinct macrophage populations identified by CD11c (CD11c^hi^ and CD11c^lo^) were detected in decidual tissues. Same as Houser’s study, we also found increased numbers and complexity of activated CD11c^hi^ Mφ in RPL patients (42). Meanwhile, the higher expression of HLA-DR^+^ and CD62L^+^CD11c^hi^ Mφ may suggest that they could participate in disease progression by higher antigen-presenting capacity and pro-inflammatory ability (43). In pregnancy, limited DCs could contribute to minimizing immunogenic and migratory DC-mediated T cell responses to feta/placental antigens (44). Unlike other studies, our data showed the number of DCs (BDCA1^+^, BDCA3^+^, pDCs) in decidual tissue was low, and no difference between HDs and RPL patients, the underlying mechanism may be the small sample size in this study. Some researchers demonstrated that the increased neutrophile ratio may be a risk factor for RPL, but the exact mechanism still needs further investigation (45). Through our investigation, the frequency of neutrophils and their subtypes were no significant differences between RPL patients and HDs. Consistent with previous findings, the ratio of other granulocytes including MCs, basophils, and eosinophils was extremely low in decidual and showed no difference in RPL patients and HDs (46).

We then explored the relationship between clinical characteristics and immune cell populations. Our results showed that the peripheral neutrophils correlated positively with T cells, supporting previous reports that the neutrophil-to-lymphocyte ratio may be a clinically predictive risk marker for RPL (45). NKT-like cells could regulate the polarization of T cells and the secretion of proinflammatory cytokines (47). In our study, we also found decidual T cells had a positive correlation with NKT-like cells, suggesting that the higher expression of NKT-like cells may disrupt the balance of T cells and promote inflammation that induces the RPL. We found that decidual CD11C^hi^ Mφ may have a positive correlation with the number of miscarriages, which suggested that the higher ratio of CD11C^hi^ Mφ may disrupt the immune tolerance at the maternal-fetal interface (48). Although positive correlations (peripheral neutrophils and decidual CD11C^hi^ Mφ) and an inverse association (peripheral T cells) between immune cells and previous miscarriages were observed in our study, the results did not reach statistical significance, probably due to the limited quantity of RPL patients. Larger prospective studies are required to confirm these findings.

Though our study has confirmed some findings and provided new data on the peripheral and decidual immune landscape of RPL patients, there were limitations to this study. First, the number of RPL samples and matched HDs was limited. Second, we have not made an in-depth analysis of different immune cell subpopulations like T and NK cells. Third, the mechanism of immune disorders of RPL patients still needs further investigation. However, using flow cytometry could simultaneously analyze multiple immune cell populations. In future studies, the detailed characterization and function of the peripheral blood and decidual immune cell composition in RPL patients should be investigated.

In conclusion, we characterized the immune cell composition in peripheral blood and decidual tissues from 23 HDs and 21 RPL patients. Our study reveals that the abnormalities of the immune system may account for an important factor leading to RPL. The multiple interactions among various immune cells may affect the balance between immune activation and clinical characteristics *via* many possible mechanisms, thus causing the development of RPL. Thus, targeting immune cell dysfunctions may provide great potential to treat RPL in clinical practice.

## Data availability statement

The raw data supporting the conclusions of this article will be made available by the authors, without undue reservation.

## Ethics statement

The studies involving human participants were reviewed and approved by Ethics Committee, Shanghai First Maternity and Infant Hospital. The patients/participants provided their written informed consent to participate in this study.

## Author contributions

XZ and SB designed and supervised the study. DQ, HX, and ZC conceived the project. XD collected clinical samples and analyzed the clinical data. HX, DQ, and SJ performed the experiments and analyzed the data. DQ and ZC wrote the manuscript. SB and XZ participated in the manuscript editing and discussion. All authors contributed to the article and approved the submitted version.

## Funding

This study was supported by the Strategic Priority Research Program (No. XDB29030302), Frontier Science Key Research Project (QYZDB-SSW-SMC036), Chinese Academy of Sciences; National Key Research and Development Program of China (2021YFE0200600); National Natural Science Foundation of China (No. 31770960), Shanghai Municipal Science and Technology Major Project (No. 2019SHZDZX02), and Shanghai Committee of Science and Technology (17411967800).

## Conflict of interest

The authors declare that the research was conducted in the absence of any commercial or financial relationships that could be construed as a potential conflict of interest.

## Publisher’s note

All claims expressed in this article are solely those of the authors and do not necessarily represent those of their affiliated organizations, or those of the publisher, the editors and the reviewers. Any product that may be evaluated in this article, or claim that may be made by its manufacturer, is not guaranteed or endorsed by the publisher.

## Supplementary material

Supplementary Figure 1 Regulated peripheral immune cell subpopulations differentially abundant between RPL patients and HDs identified by flow cytometry.

Supplementary Figure 2 Regulated decidual immune cell subpopulations differentially abundant between RPL patients and HDs identified by flow cytometry.
