# Supplementary figures and images for: The peripheral and decidual immune cell profiles in women with recurrent pregnancy loss
Source: Front Immunol. 2022 Sep 13;13:994240. doi: 10.3389/fimmu.2022.994240 (PMC9513186; doi:10.3389/fimmu.2022.994240)

# SUPPLEMENTAL FIGURE 1

A

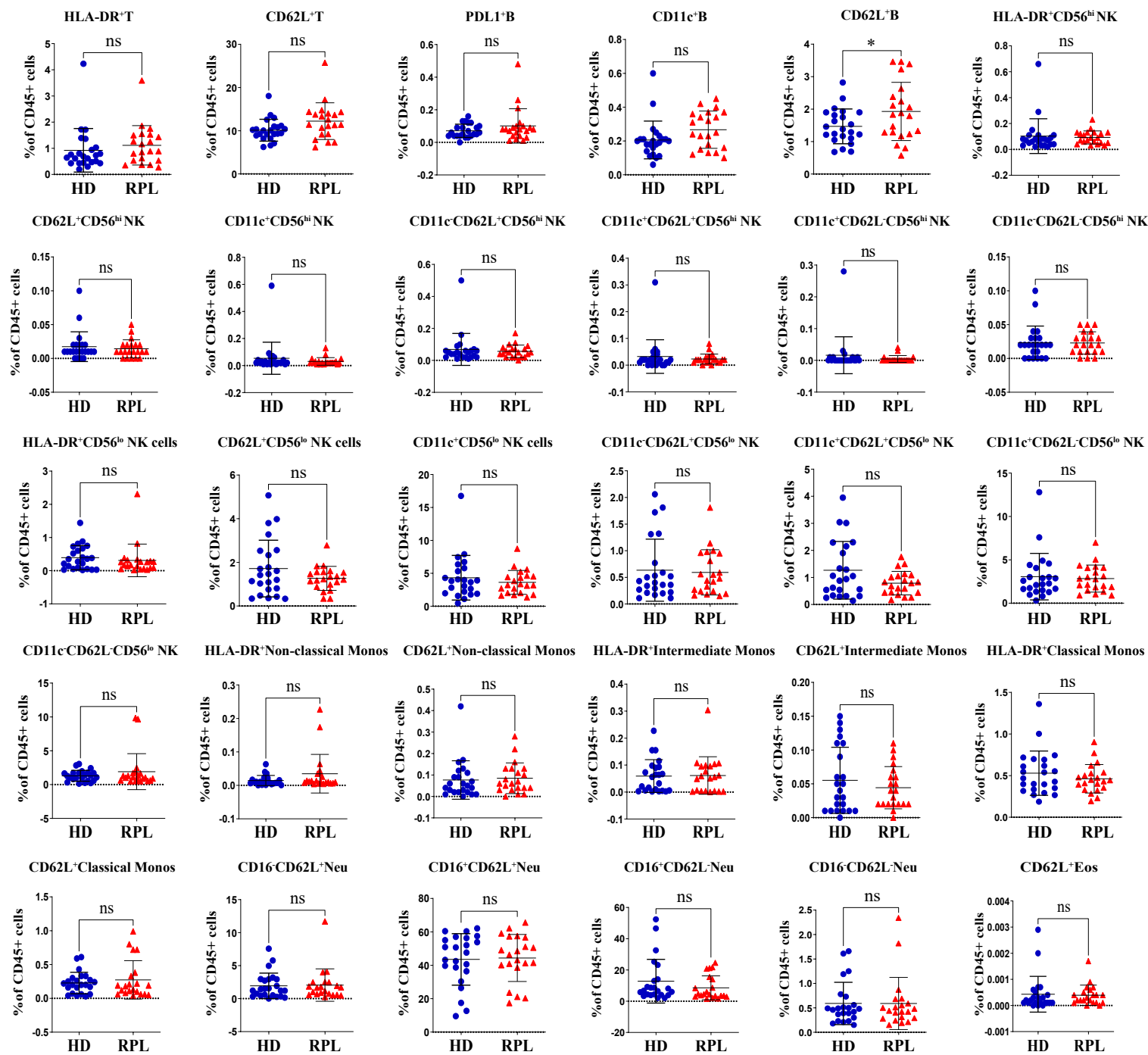

# SUPPLEMENTAL FIGURE 2

A

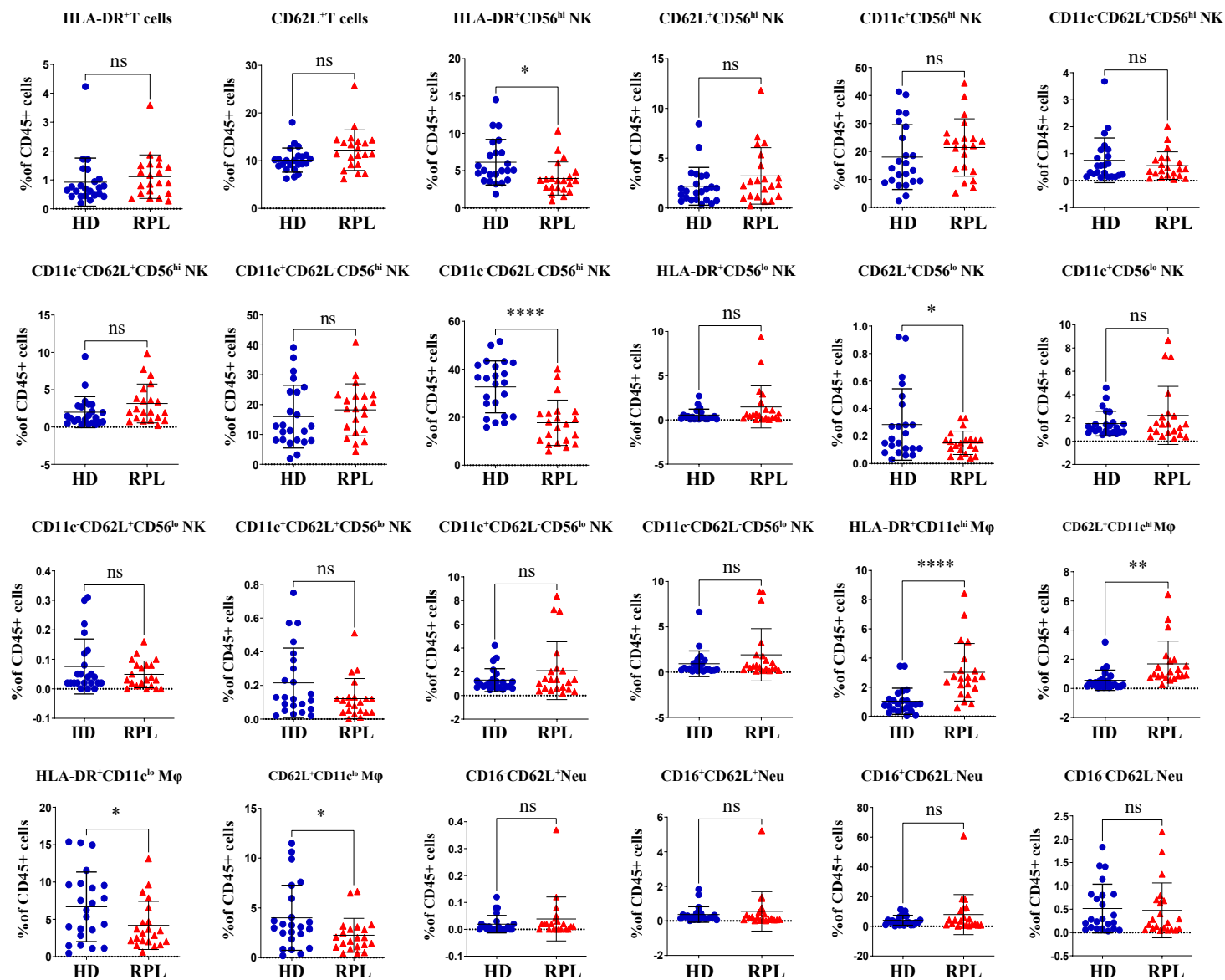

Supplement: Supplementary Figure 1 — Regulated peripheral immune cell subpopulations differentially abundant between RPL patients and HDs identified by flow cytometry. [file DataSheet_1.pdf]
