# Supplementary material for: The peripheral and decidual immune cell profiles in women with recurrent pregnancy loss
Source: Front Immunol. 2022 Sep 13;13:994240. doi: 10.3389/fimmu.2022.994240 (PMC9513186; doi:10.3389/fimmu.2022.994240)
Supplement: Supplementary file 2 [file Table_1.docx]

| Cell population % of CD45^+^cells in HD %of CD45^+^cells in RPL p value  (mean±SD) (mean±SD) |
| --- |

**Table.S1** The mean percentages of peripheral immune cells in HD and RPL patients

T cells 16.63±4.10 20.80±6.08 0.0066

NKT cells 0.06±0.05 0.23±0.22 0.0011

B cells 1.98±0.60 2.63±1.12 0.0156

NK cells 8.49±5.09 8.28±3.87 0.9254

CD56^hi^ NK cells 0.15±0.20 0.12±0.06 0.6750

CD56^lo^ NK cells 6.25±4.29 6.13±3.35 0.9059

Monocytes 3.73±1.25 3.54±1.01 0.7216

Non-classical monocytes 0.22±0.14 0.25±0.15 0.6873

Intermediate monocytes 0.18±0.10 0.16±0.08 0.3840

Classical monocytes 3.03±1.20 2.87±0.91 0.7730

pDCs 0.06±0.04 0.07±0.04 0.4748

BDCA1^+^ mDCs 0.05±0.04 0.06±0.03 0.6872

BDCA3^+^ mDCs 0.01±0.01 0.02±0.02 0.1093

Neutrophiles 60.94±8.83 56.32±9.63 0.0659

Basophils 0.27±0.16 0.27±0.17 0.8769

Eosinophiles 1.64±3.48 2.30±2.65 0.4582

| Cell population % of CD45^+^cells in HD %of CD45^+^cells in RSA p value  (mean±SD) (mean±SD) |
| --- |

**Table.S2** The mean percentages of decidual immune cells in HD and RPL patients

T cells 9.34±4.11 14.19±6.94 0.0068

NKT cells 0.23±0.15 0.79±0.65 0.0002

B cells 1.61±2.08 1.36±2.16 0.7042

NK cells 55.36±7.91 42.62±13.12 0.0003

CD56^hi^ NK cells 52.77±8.66 38.69±14.77 0.0003

CD56^lo^ NK cells 2.59±2.19 3.94±3.41 0.1235

Macrophages 10.32±5.95 10.07±4.28 0.8734

CD11C^hi^ CD14^+^Mφ 1.31±1.01 4.07±2.50 0.0001

CD11C^lo^ CD14^+^Mφ 7.73±5.18 4.68±3.47 0.0283

pDCs 0.06±0.05 0.09±0.08 0.1642

BDCA1^+^ mDCs 0.20±0.24 0.18±0.12 0.6906

BDCA3^+^ mDCs 0.81±0.53 0.75±0.42 0.6593

Neutrophiles 5.12±4.20 8.00±10.64 0.2370

Basophils 0.01±0.01 0.01±0.01 0.8250

Eosinophiles 0.05±0.05 0.07±0.08 0.3494

Mast cells 0.17±0.15 0.20±0.20 0.5237

**Table.S3.**Anti-human antibodies used in flow cytometry (FACS)

Anti-body Channel Dilutions Clone Company

CD1c PerCP5.5 1:250 RPA-2.10 eBioscience

CD123 APC 1:250 6H6 Biolegend

CD14 A700 1:500 HCD14 Biolegend

CD3 APCcy7 1:200 SK7 Biolegend

HLA-DR BV421 1:250 G46-6 BD

CD45 BV510 1:250 HI30 Biolegend

CD15 BV570 1:200 HI98 Biolegend

CD56 BV605 1:250 NCAM16.2 BD

c-Kit BV650 1:200 104D2 Biolegend

CD33 BV711 1:250 P67.6 Biolegend

CD16 BV786 1:250 3G8 BD

PDL1 PE 1:250 MIH1 eBioscience

BDCA3 PC594 1:250 M80 Biolegend

CD62L PECy5 1:250 DREG-56 invitrogen

CD19 PECy5.5 1:200 SJ25C1 eBioscience

CD20 PECy5.5 1:200 2H7 eBioscience

CD11c PECy7 1:500 Bu15 Biolegend
